# Intervention Use and Symptom Change With Unguided Internet-Based Cognitive Behavioral Therapy for Depression During the COVID-19 Pandemic: Log Data Analysis of a Convenience Sample

**DOI:** 10.2196/28321

**Published:** 2021-07-16

**Authors:** Caroline Oehler, Katharina Scholze, Hanna Reich, Christian Sander, Ulrich Hegerl

**Affiliations:** 1 Department of Psychiatry and Psychotherapy Universitätsklinikum Leipzig Leipzig Germany; 2 Forschungszentrum Depression Stiftung Deutsche Depressionshilfe Leipzig Germany; 3 Forschungszentrum Depression Stiftung Deutsche Depressionshilfe Frankfurt Germany; 4 Clinic for Psychiatry, Psychosomatics and Psychotherapy Universitätsklinikum Frankfurt Frankfurt Germany

**Keywords:** iCBT, internet-based cognitive behavioral therapy, internet-based treatment, internet- and mobile-based intervention, depression, guidance, unguided, COVID-19

## Abstract

**Background:**

Internet- and mobile-based interventions are most efficacious in the treatment of depression when they involve some form of guidance, but providing guidance requires resources such as trained personnel, who might not always be available (eg, during lockdowns to contain the COVID-19 pandemic).

**Objective:**

The current analysis focuses on changes in symptoms of depression in a guided sample of patients with depression who registered for an internet-based intervention, the iFightDepression tool, as well as the extent of intervention use, compared to an unguided sample. The objective is to further understand the effects of guidance and adherence on the intervention’s potential to induce symptom change.

**Methods:**

Log data from two convenience samples in German routine care were used to assess symptom change after 6-9 weeks of intervention as well as minimal dose (finishing at least two workshops). A linear regression model with changes in Patient Health Questionnaire (PHQ-9) score as a dependent variable and guidance and minimal dose as well as their interaction as independent variables was specified.

**Results:**

Data from 1423 people with symptoms of depression (n=940 unguided, 66.1%) were included in the current analysis. In the linear regression model predicting symptom change, a significant interaction of guidance and minimal dose revealed a specifically greater improvement for patients who received guidance and also worked with the intervention content (*β*=–1.75, *t*=–2.37, *P*=.02), while there was little difference in symptom change due to guidance in the group that did not use the intervention. In this model, the main effect of guidance was only marginally significant (*β*=–.53, *t*=–1.78, *P*=.08).

**Conclusions:**

Guidance in internet-based interventions for depression is not only an important factor to facilitate adherence, but also seems to further improve results for patients adhering to the intervention compared to those who do the same but without guidance.

## Introduction

When the COVID-19 pandemic hit Germany in spring 2020, many patients with depression suddenly lost their access to care [1]. Face-to-face appointments were cancelled, self-help groups could not meet and planned or started inpatient treatments were suspended. In this situation, great expectations were placed on internet- and mobile-based interventions (IMIs). IMIs have repeatedly been proven efficacious in the treatment of depression [2]. In particular, when they are implemented with some form of professional guidance, the benefits for patients with depression are substantial [3] and might even reach the level obtained with face-to-face interventions [4].

Although evidence suggests that guided IMIs should be preferred to unguided ones [5], limited resources of the health care system or other circumstances often prevent professional guidance from being provided. Previously, smaller effect sizes in unguided interventions have been explained in part by lower adherence. Lower adherence, in turn, has been associated with reduced treatment effects [6]. However, it is possible that guidance also enhances intervention effects in other ways, such as improving treatment credibility [7] or deepening understanding of the material, but this relationship has not been conclusively established. In order to fully utilize the possibilities of IMIs, it is in turn necessary to understand factors contributing to their efficacy and effectiveness.

One of these guided programs is the iFightDepression tool (iFD). It is based on cognitive behavioral therapy (CBT) techniques and its effectiveness has been demonstrated in comparison to an active control group [8]. Usually, people with depressive disorders receive access to iFD only through their physician or psychotherapist, who then also provides guidance. Guidance is meant mainly to encourage users and to help when questions or difficulties with the material arise. Users can decide if they want to share the entries they make in iFD with their guides. They are advised to work through the six core workshops in 6-12 weeks, but access to iFD is not limited to a certain time span. Due to significantly increased demand coinciding with the limited accessibility of health care providers during the first months of the COVID-19 pandemic, interested persons could contact the iFD team directly and receive unguided access to the tool from March to June 2020. This initiative was publicized in a press release, which was picked up by various digital and print media as well as by radio stations. An announcement was also posted on the home page of the Stiftung Deutsche Depressionshilfe and was met with great interest.

This situation provides a natural laboratory to compare user behavior and self-reported symptoms from a guided sample in routine care to an unguided sample. This short paper assesses the following questions:

1. Does the change in symptoms of depression differ between guided and unguided users of an IMI in routine care and is this related to use of the intervention when controlling for the available covariates?
2. Are there factors such as concomitant treatment, age, or gender that could explain possible differences concerning changes in depressive symptoms?

## Methods

### Sample

The current analysis used routinely collected log data from users of the iFightDepression tool (iFD), a web-based CBT intervention [9] for patients with depression. Data were extracted for patients who were at least 18 years old and had given informed consent to participate in the ongoing evaluation of the tool, as is mandatory at registration; had at least minimal symptoms of depression (indicated by a score greater than 4 on the Patient Health Questionnaire [PHQ-9]); and logged onto the tool at least twice. Anonymized log data from all regular, guided accounts of the German version of iFD were used (collected from October 2016 to October 2020). These patients had received access to iFightDepression through their general practitioner, their psychotherapist, or another physician who then also provided guidance. Equivalent log data were extracted for all users who had registered for the unguided version (March-June 2020). The latter received no personalized guidance whatsoever but instead got automated weekly reminder emails for the first 6 weeks of intervention use. Those emails contained encouragement to continue using the intervention and commented on key learnings, one workshop at a time. They were sent regardless of workshop participation or completion.

### Measures

During the registration process, participants filled in a questionnaire that yielded basic sociodemographic information (age, gender, current and past treatment). As a brief measure of depression severity, the PHQ-9—a short, well-validated, and widely used measure—is integrated for self-monitoring purposes [10,11]. The PHQ-9 is mandatory at the beginning and can be filled in again at any time during the intervention. Once per week, patients are prompted to fill in the questionnaire to monitor their symptoms. Sum scores of the PHQ-9 as filled in by the users during their work with iFD were used in the current analysis.

Anonymized log files were used to study symptom development and user behavior. These log files included time-stamped logs of all activities within the iFD tool. From those, a composite measure for usage was generated. Each user who completed at least two workshops (by reading at least 70% of the texts) within the first 6 weeks was regarded as having received a minimal dose of treatment that is potentially effective. This definition was chosen based on the findings that even the use of individual components of CBT can lead to reductions in symptoms of depression [12-14]. It is reasonable to assume that after completing at least two of the six workshops, patients will have learned about some CBT techniques that have the potential to alleviate their symptoms.

### Statistical Analysis

For this analysis, only data from participants who completed the PHQ-9 at least once at 6-9 weeks after enrollment were considered. Baseline differences in sociodemographic variables were tested for statistical significance using chi-square tests for categorical variables, analysis of variance for normally distributed numeric data, and Wilcoxon rank-sum tests in case of nonnormally distributed data. The *P* values were corrected for multiple testing using a false discovery rate correction [15].

The routinely collected PHQ-9 scores were used as an estimate for real-world effectiveness. As an estimate of symptom change, the mean difference (delta) of PHQ-9 scores at registration and after 6-9 weeks was calculated for all patients with available entries in the category after 6-9 weeks. This time span was used based on experience from a previous study [8], according to which effects of the intervention were the largest after the sixth week. If patients had filled in PHQ-9 more than once 6-9 weeks after their registration, a mean value was used. Since this difference score fulfilled the criteria of normal distribution, it was used as a dependent variable for a linear regression model.

A multiple linear regression model predicting delta‑PHQ was specified, including the following variables as independent variables: baseline PHQ-9, current psychotherapy (yes versus no), current antidepressant medication (yes versus no), diagnosed depression (yes versus no), age, gender, guidance (guided versus unguided), and minimal dose (achieved versus not achieved), as well as the interaction of guidance and minimal dose. This interaction coefficient is of special interest, since differences in reductions of symptoms of depression that depend both on minimal dose and group can be regarded as an estimate for a differential treatment effect between guided and unguided use. The fit of this model was compared to a reduced version without guidance and minimal dose using analysis of variance. This allows us to test if the reduction in the residual sum of squares due to the additional variables is statistically significantly different from zero. This statistical approach was chosen to control for the influence of the available baseline variables that might differ between the two groups and yield the best possible estimate for the interaction of guidance and minimal dose.

All statistical analyses were performed using R (version 3.5.1) [16] and the level of statistical significance was set at α=.05.

## Results

### Sample

On October 16, 2020, data were extracted for 9730 participants, of whom 2181 (22.42%) had been invited to use iFD by a guiding health care professional and 7549 (77.58%) had received access through unguided accounts during the first nationwide lockdown in Germany during the COVID-19 pandemic. In total, 483 (22.15%) people in the guided sample and 940 (12.45%) people in the unguided sample provided PHQ-9 data after 6-9 weeks and were included in the current analysis. Sociodemographic data broken up for guidance (yes versus no) and achieved minimal dose (yes versus no) are displayed in Table 1. The proportion of users achieving minimal dose did not significantly differ between guided and unguided users (*X*^2^_1423_=2.8846, *P*=.09).

**Table 1:** Overview of sociodemographic characteristics. *P* values correspond to the comparison of participants who used iFightDepression with and without guidance.

Variables	Total (n=1423)	Guided (n=483)			Unguided (n=940)			*P* value, comparison of guided versus unguided sample (false discovery rate–adjusted *P* value)
		Minimal dose achieved (n=66)	No minimal dose (n=417)	Minimal dose achieved (n=158)		No minimal dose (n=782)		
Female, n (%)	946 (66.45)	42 (63.64)	266 (63.79)	106 (67.09)		533 (68.16)	.11 (.13)	
Age in years, mean (SD)	40.15 (13.35)	38.77 (13.16)	39.77 (14.13)	39.79 (12.31)		40.54 (13.14)	.26 (.26)	
Baseline Patient Health Questionnaire score, mean (SD)	14.11 (4.89)	14.68 (4.91)	14.65 (4.93)	13.29 (4.65)		13.94 (4.88)	.003 (.006)	
Diagnosed depression, n (%)	1093 (76.81)	58 (87.88)	375 (89.93)	108 (68.35)		552 (70.59)	<.001 (<.001)	
Current psychotherapy, n (%)	660 (46.38)	43 (65.15)	263 (63.07)	58 (36.71)		296 (37.85)	<.001 (<.001)	
Current antidepressant treatment, n (%)	582 (40.90)	31 (46.97)	221 (53.00)	47 (26.75)		283 (36.19)	<.001 (<.001)	
Past psychotherapy, n (%)	620 (43.57)	20 (30.30)	147 (35.25)	74 (46.84)		379 (48.47)	<.001 (<.001)	
Past antidepressant treatment, n (%)	471 (33.10)	17 (25.76)	127 (30.46)	52 (32.91)		275 (35.17)	.07 (.09)	

**Figure 1:**
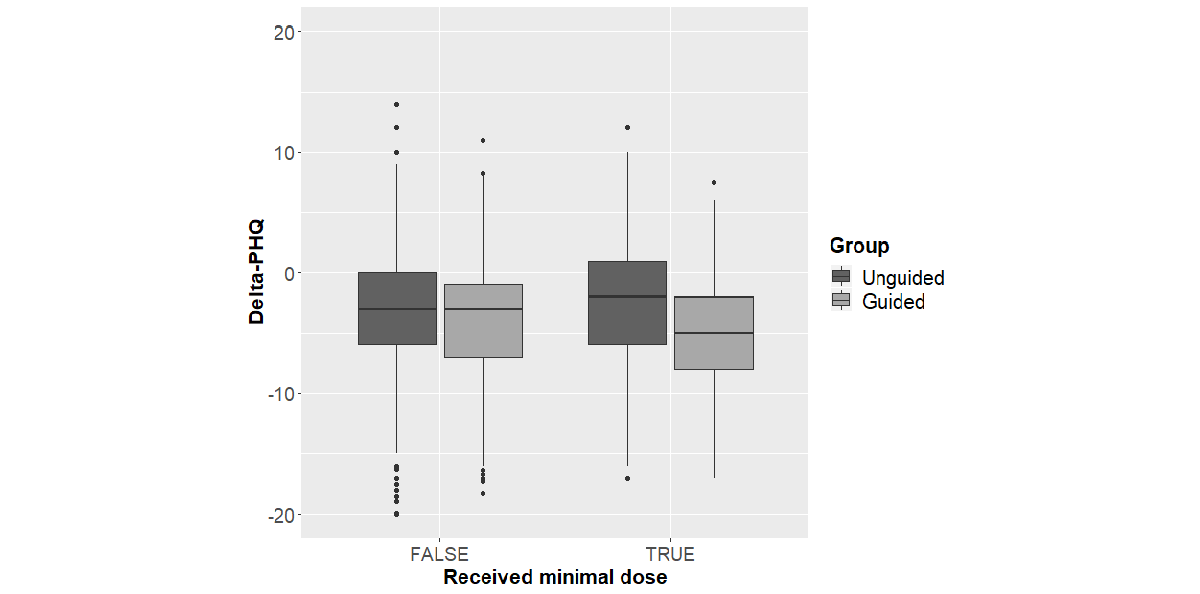
Boxes indicate the 25th to 75th percentile, while the bold line in the box indicates the mean delta-PHQ. Whiskers mark the largest value within 1.5 times interquartile range above the 75th percentile and below the 25th percentile. Values further out are marked as points and are potential outliers. Delta-PHQ refers to the change in participant score on the Patient Health Questionnaire.

Results of the multiple linear regression predicting delta-PHQ indicated a statistically significant interaction of guidance and minimal dose (*β*=–1.75, *t*=–2.37, *P*=.02; Figure 1), while the main effects of guidance (*β*=–.53, *t*=–1.78, *P*=.08; Table 2) and minimal dose (*β*=.51, *t*=1.27, *P*=.21) did not reach significance. A larger reduction in symptoms of depression was also associated with a greater PHQ-9 score at baseline (*β*=–.42, *t*=–15.84, *P*<.001), being younger (*β*=–.02, *t*=–2.31, *P*=.02), and not reporting being diagnosed with depression (*β*=.94, *t*=2.76, *P*=.006). The model estimates for nonsignificant predictors were as follows: gender (*β*=.12, *t*=0.45, *P*=.65), current psychotherapy (*β*=–.02, *t*=–0.08, *P*=.94), and current antidepressant medication (*β*=–.536, *t*=–1.88, *P*=.06). The full model led to *R*^2^=0.168 and explained significantly more variance than a basic model without guidance and minimal dose (*F*_3,1414_=4.60, *P*=.003).

**Table 2:** Differences in Patient Health Questionnaire scores at baseline and after 6-9 weeks of program use.

Sample	Patient Health Questionnaire score at start of intervention, mean (SD)	Patient Health Questionnaire score after 6-9 weeks intervention, mean (SD)	Within-group effect size, *d* (95% CI)
Guided sample (n=483)	14.65 (4.92)	10.49 (5.26)	0.82 (0.69-0.95)
Unguided sample (n=940)	13.83 (4.85)	10.72 (5.66)	0.59 (0.50-0.68)

## Discussion

### Principal Findings

This retrospective analysis of a convenience sample provides a new perspective on the importance of guidance in IMIs. While both guided and unguided users reported a reduction in symptoms of depression, the differences varied depending on the use of the intervention. Those patients who did not interact with the intervention material (ie, completed less than two workshops) and just filled in the symptom questionnaire reported a similar reduction in symptoms of depression, independent of receiving guidance or not. On the other hand, when patients did engage with the intervention material, the reduction in symptoms of depression was superior in the guided group, as indicated by the significant interaction in the linear model. This effect was stable when other covariates that influenced delta-PHQ were taken into account (eg, baseline PHQ-9, age, and reporting being diagnosed with depression).

It needs to be mentioned that contrary to earlier results that associate adherence with effectiveness [17], in the current data set, there was no significant main effect of minimal dose. In the current sample, minimal dose was only predictive through its interaction with guidance.

One interpretation of this interaction could be that the role of guidance goes beyond just facilitating adherence. It might be that the guided version of iFD was perceived as more credible or that guides could help in correcting misinterpretations concerning the exercises. A further possible explanation is that patients working through the program might take the tasks more seriously when they know that a professional is guiding them and taking care of them. Both would enhance the use of CBT techniques and might create a greater positive expectation, strengthening the placebo effect. Still, it is also possible that patients with more persistent symptoms during the 6-9 weeks of data collection had a harder time motivating themselves to engage with the intervention when unguided and therefore achieved the minimal dose less often.

Concerning the covariates, the current results only partly replicate existing knowledge. Higher symptom scores before the intervention have been regularly associated with greater reductions during treatment [18], possibly because of regression to the mean as well as floor effects for patients with initially mild symptoms.

Although results concerning both age and gender as predictors for adherence/effectiveness have been inconclusive in a meta-analysis [19], another trial of a large sample of community users in Australia showed higher engagement with an intervention for younger users [20] and thus points in a similar direction as the current analysis.

Finally, the association of not being diagnosed with depression with greater improvement might be a sign that (especially in the unguided group) some patients might have had symptoms of depression caused by acute stressors without fulfilling the criteria of major depression. That could lead to a faster decline in symptoms of depression in this group but this is only speculative at this stage.

It is noteworthy that the amount of variance in delta-PHQ that can be explained by the current model is rather low (16.8%). Although changes in symptoms of depression will naturally depend on many situational and intrapersonal variables, it is possible there might be other relevant variables influencing the changes in delta-PHQ that were not assessed in the current analysis (eg, attitudes toward online interventions, expectations of success).

### Limitations

When interpreting these results, it needs to be kept in mind that no randomization was applied and the selection process between guided and unguided samples clearly differed. In order to assess the impact of the limitations described above on the validity of the study, it is useful to examine where exactly the risks lie. Although the guided group had been invited by their treating health care professional, the unguided group was self-selected. Self-selection occurred during the first weeks of the COVID-19 pandemic. This might covary with participant characteristics (eg, depressive symptoms) in view of acute stress due to lockdown measures versus participants with depressive disorders treated in care as usual by the guides. Although the current analysis controlled for baseline differences in the variables that were available, there might be differences between the two samples in other areas. It is possible that the unguided sample had more comorbid disorders and was therefore less likely to benefit from a CBT intervention for depression or that the pandemic situation made it more difficult to actually use the CBT techniques and therefore the minimal dose was still not effective. In addition, the amount or content of guidance is not documented or known in this context, so it is possible that there was a large variance in this. Finally, the mean number of days between first and last login for the current sample was 27, so the percentage of people who provided data after 6-9 weeks was low. This analysis therefore only considers those users who for some reason logged into the tool again after that time span and filled in the PHQ-9; it cannot be known if this subgroup was representative for all users (for details, see Multimedia Appendix 1). This finding underscores the need to design interventions in a way that supports adherence and user engagement [21,22]. The results on the interaction of guidance and minimal dose should therefore be regarded as preliminary and should be followed up on in randomized trials.

### Conclusions

This analysis yields some further evidence that guidance is an essential part of IMIs targeting depression. It is known from past research that guidance has a positive effect on adherence; in the current data set, it is associated with greater improvements in symptoms of depression. This underscores the importance of creating conditions in the health care system to provide IMIs with professional guidance. Although many patients spontaneously provided feedback of being very grateful for this low-threshold intervention, based on this analysis, the strategy seems less appropriate. In comparable situations where regular care pathways are not available, unguided digital interventions for depression should therefore not be the preferred and only option.

## Appendix

Multimedia Appendix 1 Overview of sociodemographic characteristics. <italic>P</italic> values correspond to the comparison of participants who did versus did not provide Patient Health Questionnaire data after 6-9 weeks.

## Abbreviations

CBT: cognitive behavioral therapy
EAAD: European Alliance Against Depression
iFD: iFightDepression
IMI: internet- and mobile-based intervention
PHQ-9: Patient Health Questionnaire
