# Supplementary material for: Intervention Use and Symptom Change With Unguided Internet-Based Cognitive Behavioral Therapy for Depression During the COVID-19 Pandemic: Log Data Analysis of a Convenience Sample
Source: JMIR Ment Health. 2021 Jul 16;8(7):e28321. doi: 10.2196/28321 (PMC8288646; doi:10.2196/28321)
Supplement: Multimedia Appendix 1 [file mental_v8i7e28321_app1.pdf]

Multimedia Appendix 1

Unguided iCBT for depression during the COVID-19 pandemic - Intervention usage and symptom change in a convenience sample compared to guided use in regular care

Authors: Oehler C, Scholze K, Reich H, Sander C, Hegerl U

Overview of sociodemographic characteristics. *P*-values correspond to the comparison of participants who did vs did not provide PHQ-9 data after 6-9 weeks.

Variables	Provided PHQ-9 after 6-9 weeks (n= 1423)	No PHQ-9 after 6-9 weeks (n=8307)	*P* – comparison (FDR adjusted *P*)
Female - n (%)	946 (66.45%)	5713 (68,78%)	.092 (.148)
Age in years (SD)	40.15 (13.35)	38.14 (12.45)	<.001 (<.001)
Baseline PHQ-9	14.11 (4.89)	14.38 (4.88)	.035 (.071)
Diagnosed depression - n (%)	1093 (76.81%)	5896 (70.76%)	<.001 (<.001)
Currently PT - n (%)	660 (46.38%)	3389 (40.80%)	<.001 (<.001)
Currently AD - n (%)	582 (40.90%)	3240 (39.00%)	.184 (.211)
Past PT- n (%)	620 (43.57%)	3805 (45.80%)	.126 (.167)
Past AD- n (%)	471 (33.10%)	2850 (34.31%)	.392 (.392)

Abbreviations. PHQ-9: patient health questionnaire, SD: standard deviation, PT: psychotherapy, AD: antidepressant treatment, FDR: false discovery rate.
